# Bridging assessment and treatment for repeat suicidality in prisons: development and validation of a risk model

**DOI:** 10.1136/bmjment-2024-301280

**Published:** 2025-02-27

**Authors:** Seena Fazel, Leanne Heathcote, Leen Farouki, Jane Senior, Amanda Perry, Thomas R Fanshawe, Jenny Shaw

**Affiliations:** 1 Department of Psychiatry, University of Oxford, Oxford, UK; 2 Oxford Health NHS Foundation Trust, Oxford, UK; 3 Division of Psychology and Mental Health, University of Manchester, Manchester, UK; 4 Health Sciences, University of York, York, UK; 5 Nuffield Department of Primary Health Care Sciences, University of Oxford, Oxford, UK

**Keywords:** Suicide & self-harm, Forensic psychiatry

## Abstract

**Background:**

Suicidal thoughts and behaviours are common in people in prison and associated with poor health outcomes, including suicide, injury and repeat self-harm.

**Objective:**

To develop and validate a model to stratify risk of repeat suicidality up to 3 months in people in prison.

**Methods:**

In seven English prisons, we identified 754 people aged over 17 who had been placed on a suicide risk management plan after a self-harm episode or elevated risk. We developed a multivariable model to stratify risk of repeat suicidality at 3 months using routinely collected sociodemographic, clinical and prison-related factors, which were tested using Cox proportional HR models. In a prospective validation sample of 390 people from 13 prisons, we tested this model to assess risk of repeat suicidality at 3 months across a range of performance measures.

**Findings:**

Of the overall sample of 1144 people in prison (n=966 men or 84%, mean age 33 years), 22% had the outcome of repeat suicidality over 3 months. The final risk model consisted of nine factors, including sex, calendar age and features of recent suicidal behaviour. Calibration and discrimination were similar in both development and validation samples, with O:E ratio=1.09 (95% CI 0.88 to 1.35) and c-statistic=0.66 (95% CI 0.60 to 0.72) in external validation. At a 25% cut-off, sensitivity was 58% (50 to 66) and specificity was 72% (68 to 75) in external validation. The tool (Risk Assessment for people in Prison at risk of Self-harm and Suicide, RAPSS) is available as an online risk calculator at https://oxrisk.com/rapsstrial/.

**Interpretation:**

A novel assessment approach for repeat suicidality can provide an evidence-based approach to stratify risk and better allocate resources.

**WHAT IS ALREADY KNOWN ON THIS TOPIC**

- Repeat self-harm is common in people in prison, and there are no risk assessment tools or risk models that can inform management of risk for individuals in prison who have self-harmed. Currently, unstructured approaches by prison (and occasionally healthcare) staff are typically how risk is assessed and who receives treatment, and decisions about the allocation of resources are not informed by risk.

**WHAT THIS STUDY ADDS**

- A simple risk model comprising of nine individual-level factors can accurately identify repeat suicidality in people in prison. Some of these factors have not been linked with this risk previously, underscoring the approach taken and large sample used.

**HOW THIS STUDY MIGHT AFFECT RESEARCH, PRACTICE OR POLICY**

- A scalable and transparent risk model, translated into a freely available online calculator (Risk Assessment for people in Prison at risk of Self-harm and Suicide, RAPSS), can usefully inform the risk management of people in prison at risk of repeat suicidality. Future research can consider whether its implementation improves adverse outcomes.

## Introduction

 Rates of self-harm in people in prison are high in absolute and relative terms. In 2023, in England and Wales, there were 70 875 reported incidents of self-harm in more than 12 000 people in prison, which has increased from around 23 000 incidents in 2012–2013.^1^ Over the same period, there were 3349 hospital attendances from prison because of self-harm incidents, a substantially higher rate than people in the community.^2^ Internationally, self-harm is a leading cause of morbidity for people in prison^3^, and strongly associated with the high suicide rates during custody^4^ and on release.^5^

Current practice in many high-income countries is to initiate a suicide risk management plan^6^ for people in prison presenting with suicidal ideation or who have self-harmed. In England and Wales, this risk management plan is known as an ‘Assessment, Care in Custody and Teamwork’ (ACCT), which can be opened by any staff member. Typically, a prison officer opens and also completes it, who receive training on suicide risk assessment as part of their induction and subsequent refresher courses. The ACCT process involves setting out an immediate plan that focuses on safety, followed by a series of multidisciplinary meetings to consider the observation level and nature of additional support the individual requires. A ‘Care Map’ should be drawn up at the first multidisciplinary meeting, which involves an ongoing action plan outlining how care and support are delivered, over what time and by whom. The ACCT can be closed when the multidisciplinary team come to the view that the care plan is addressed, risk reduced and it is judged safe to close it. These decisions are subjective—there is no structured approach to inform when to close.

Although this approach may be effective in managing the immediate risk (while the management plan lasts), risk of repeat self-harm has been shown to be high in the few weeks after closure of the plan,^6^ and there is no empirical evidence that has shown that having been under the ACCT process reduces repeat suicidality and self-harm^7^ or suicide rates. In addition, there are no structured approaches for the assessment of risk of repeat self-harm despite high rates. One UK study reported 28% of people in prison self-harmed in the 6 months following ACCT closure.^8^ Additionally, the mean number of self-harm incidents per self-harming individual is around 5–6 over a 12-month period.^1^

Repeat self-harm has significant implications for public health and safety. Alongside physical injuries arising from self-harming behaviour, there is the potential for psychological distress of staff,^9 10^ other people in prison and their families. More serious injuries consume additional resources due to liaison with external hospitals and psychiatric services,^11^ which can in turn impact on the stability of the prison environment for rehabilitation. Thus, scalable, valid and feasible approaches to stratify risk of further suicidality can lead to resource savings^12^ and improve consistency of assessments within and between services.^13^ Despite this, clinical guidelines in England have recommended a shift away for simplistic checklists to assess risk, and new approaches should address critiques of previous tools.

In this study, we describe the development and external validation of a risk model for repeat suicidality within 3 months following the closure of an ACCT suicide risk management plan.

## Methods

### Study design

We conducted a retrospective cohort study of people in English prisons who were placed on an ACCT risk management plan (see online supplemental appendix for analysis plan). Eligible individuals were aged at least 18 years who had an ACCT closed in the previous 18 months and were detained in seven male and female prisons across northern England. To develop the model, data collection started in December 2021 (for index ACCTs from October 2021), with follow-up information obtained to ensure 90 days follow-up per person. The index ACCT was the first ACCT during the study period in each eligible person, who made up the development sample. Data collection concluded in 2023. The outcome was defined as the time of opening of repeat ACCT within 90 days after closure of index ACCT. This included repeat self-harm episodes, and people considered at risk of self-harm or suicide (including an escalation in self-harm, suicidal thoughts and/or low mood), which we have termed ‘repeat suicidality’. This outcome is broader than self-harm and captures one with relevant impacts on clinical and prison services. There was no censoring for other reasons (apart from repeat ACCT or end of follow-up) as participants were required to have at least 90 days of follow-up for inclusion. These data were collected by research assistants who were qualified to at least undergraduate level and specifically trained for the project, and who liaised with prisons to extract information on risk factors and outcomes (see below).

A validation sample was obtained using a different time period (January 2023 to June 2023) and recruited from five of the prisons used in the development sample and adding a further eight from London and South and West of England (making a total of 13 prisons). No individuals as part of the external validation were in the development sample. Research assistants liaised with individual prisons to directly collect the same information on risk factors and outcomes as per the development sample. Predictions in the validation sample were calculated using the equation of the development model sample, with same discrimination and calibration measures as in development sample.

### The ACCT process

The ACCT document is a series of forms held together in a folder. It is ‘opened’ by staff working in prisons in response to a self-harm episode, low mood or concerns that an individual is at risk of self-harm or suicide. On closure, when the risk is deemed no longer present, the ACCT document can be reopened during the next 7 days with further concerns, and a postclosure assessment is completed after 7 days.

### Risk factors

Potential risk factors comprising sociodemographic information, clinical history and treatment, and criminal records were obtained from the electronic health records (‘SystmOne’), prison records (‘C-NOMIS’ [Computer-National Offender Management Information System]) and ACCT documents. These included a core set (sex at birth, age, reason for ACCT opening, and previous ACCT within 6 months before index ACCT) based on population-based studies of self-harm in English and Welsh prisons^14^ and systematic reviews.^3 15^ In addition, we tested other factors based on this previous research (eg, method of self-harm^14^ and diagnosed psychiatric conditions, substance misuse, index violent crime^15^) for whether they could improve predictive performance of the multivariable model (see online supplemental appendix for analysis plan). Candidate risk factors available in the datasets were allocated into two groups *a priori* (see Protocol; for details, see online supplemental appendix p.12), and those in group 2 selected using backward stepwise selection (5% significance level). Researchers worked independently, and as a measure to minimise bias and maximise sample validity, the study team completed 10% sample checks.

### Statistical analysis

We fitted a Cox proportional HR, with the time of ACCT reopening as the outcome event (details in online supplemental appendix for analysis plan). There was no evidence against the proportional hazards assumption. The model was used to calculate the probability of ACCT reopening within 90 days follow-up using the estimated baseline survival. Target sample size considerations included>10 outcome events (ie, repeat ACCTs) per predictor for development, and>100 outcome events for validation. Internal validation to adjust for possible overfitting used bootstrapping with 1000 replicates, and model performance was summarised using bootstrapped estimates of predictive accuracy including Harrell’s c-index for time-to-event data and estimates of sensitivity and specificity corresponding to different risk thresholds (from 15%–35% in 5% increases, based on expected prevalence of repeat suicidality). The proportions of predicted and observed events at different predicted probabilities were compared using a calibration plot.

Predictions in the validation sample were calculated using the equation of the development model. TRIPOD guidelines were followed for design and reporting^16^ (see online supplemental file 2). We created a web-based risk calculator to easily calculate predicted probabilities. Analysis was performed using R version 4.2.1.

## Results

### Development sample and characteristics

We assessed 990 people in prison, and after removing duplicates and those without follow-up data, the development sample was 754 individuals, from seven prisons (range 74 to 153 people/prison; six male prisons and one female prison; online supplemental eFigure 1 for flowchart). Index ACCTs occurred between April 2016 and March 2023, with the majority in 2021 and 2022.

Most participants were male (88%), which was similar to all people in the included prisons, and median age was 34 years (range 17–79 years) (see table 1 for risk factor information for the full primary analysis cohort and split by outcome). Cutting was the predominant method of self-harm. In relation to background factors, 58% had a history of self-harm at screening on arrival into custody and 31% had a previous ACCT in the 6 months before the index ACCT. A majority of the sample were prescribed medication at the time of the index ACCT, with 53% on antidepressants and 23% on antipsychotics.

**Table 1:** Development sample characteristics of people in prison on a suicide risk management plan (ACCT)

Risk factor	Total(n=754)	No follow-up ACCT within 90 days(n=587)	Repeat ACCT within 90 days(n=167)
Male sex	661 (88%)	522 (89%)	139 (83%)
Age (years)	34 (28, 44)	35 (28, 45)	33 (26, 39)
**Reason for ACCT opening**			
Low mood	211 (28%)	180 (31%)	31 (19%)
Threat of self-harm	239 (32%)	188 (32%)	51 (31%)
Self-harm	304 (40%)	219 (37%)	85 (51%)
Suicidal thoughts (at ACCT opening)	346 (46%)	265 (45%)	81 (49%)
Previous ACCT within 6 months before index ACCT	232 (31%)	162 (28%)	70 (42%)
Ethnicity: non-white	177 (28%)	146 (30%)	31 (21%)
Marital status: currently single	468 (90%)	353 (90%)	115 (91%)
Violent index offence	556 (74%)	429 (73%)	127 (76%)
First time in custody	345 (46%)	286 (49%)	59 (35%)
**Sentence type**			
Life	71 (9%)	59 (10%)	12 (7%)
IPP/other*	104 (14%)	81 (14%)	23 (14%)
Time between ACCT closure and expected release from prison (years)	1.8 (0.3, 3.9)	1.8 (0, 4.0)	1.8 (0.4, 3.5)
Drug screen	288 (38%)	212 (36%)	76 (46%)
Time between reception into custody and ACCT opening (days)	287 (24, 1148)	264 (24, 1050)	326 (24, 1738)
**Self-harm method** †			
Cutting	167 (22%)	113 (19%)	54 (32%)
Strangulation	45 (6%)	33 (6%)	12 (7%)
Overdose	55 (7%)	44 (7%)	11 (7%)
Other method	33 (4%)	24 (4%)	9 (5%)
CAREMAP completed	544 (76%)	414 (74%)	130 (82%)
Physical health/GP referral	190 (25%)	141 (24%)	49 (30%)
Mental health referral	350 (47%)	263 (45%)	87 (52%)
IDTS/DARS referral	59 (8%)	45 (8%)	14 (8%)
Raised/High risk at first case review	97 (13%)	74 (13%)	23 (14%)
Friend/family support	655 (89%)	504 (88%)	151 (94%)
**Previous diagnoses** [Table-fn T1_FN6]			
Chronic physical condition	216 (29%)	166 (28%)	50 (30%)
Learning disability/neurodevelopmental	74 (10%)	50 (9%)	24 (14%)
Disorder			
Mental illness	62 (8%)		18 (11%)
Substance misuse	37 (5%)		9 (5%)
**Current medication**			
Antidepressants	400 (53%)	311 (53%)	89 (53%)
ADHD medication	25 (3%)	17 (3%)	8 (5%)
Antipsychotics	170 (23%)	122 (21%)	48 (29%)
Mood stabilisers	93 (12%)	72 (12%)	21 (13%)
Opioids	91 (12%)	70 (12%)	21 (13%)
Sleepers	15 (2%)	12 (2%)	3 (2%)
Pain relief	199 (26%)	151 (26%)	48 (29%)
Previous psychotropic medication (6 months before ACCT)	436 (58%)	332 (57%)	104 (63%)
Abnormal liver function enzymes	238 (32%)	178 (31%)	60 (37%)
Engaging with primary care	614 (82%)	472 (81%)	142 (86%)
Engaging with mental healthcare	632 (85%)	490 (84%)	142 (86%)
**Previous self-harm recorded at reception screening**	429 (58%)	317 (55%)	112 (68%)
In community	138 (18%)	113 (19%)	25 (15%)
In custody	90 (12%)	68 (12%)	22 (13%)
In community and in custody	201 (27%)	136 (23%)	65 (39%)
**Length of ACCT**			
0–1 days	189 (25%)	167 (28%)	22 (13%)
2–9 days	212 (28%)	166 (28%)	46 (28%)
10+ days	353 (47%)	254 (43%)	99 (59%)

Table shows n (%) or median (inter-quartile range). Percentages calculated among individuals with available data. CAREMAP=central document of ACCT.

Variables in **bold face** were pre-specified for inclusion in the risk prediction model.

Note: variables with missing data: Age: 7, Suicidal thoughts: 4, Ethnicity: 123, Marital status: 236, Index offence: 1, First time in custody: 5, Time between reception into custody and ACCT opening: 33, CAREMAP completed: 37, Physical health/GP referral: 3, Mental health referral: 3, Risk at first case review: 24, IDTS/DARS referral: 3, Friend/family support: 20, Previous psychotropic medication: 8, Abnormal liver function enzymes: 12, Engaging with primary care: 8, Engaging with mental health carehealthcare: 8, Previous self-harm recorded at reception screening: 8.

* Includes Imprisonment for Public Protection (IPP) and other non-life sentences for which no release date is specified.

† Among those with self-harm as the reason for ACCT opening. Individuals may have more than one reason for opening recorded.

‡ Chronic Physical Condition and Learning Disability / Neurodevelopmental Disorder: lifetime diagnosis. Mental Illness and Substance Misuse: diagnosis within 12 months months prior to ACCT closure.

ACCTAssessment, Care in Custody and TeamworkADHDattention deficit hyperactivity disorderDARSDrug and Alcohol Rehabliitation ServicesGPGeneral PractitionerIDTSIntegrated Drug Treatment System

The distributions of time between ACCT closure and expected prison release date, and time between arrival into custody and ACCT opening were highly positively skewed with many people in prison having an ACCT opened soon after arrival, so both variables were log transformed prior to further analysis (in order to meet normality assumptions of the statistical model).

The duration of the ACCT was also highly variable, with 25% of participants having an ACCT length of 0–1 days, and 53% less than 10 days, so this variable was recoded into three categories (table 1). ACCTs that were opened due to self-harm were more likely to last >10 days (online supplemental eTable 1).

### Outcome information

Around a fifth (n=167, 22%) of the development sample had the outcome of repeat suicidality within the first 30 days after index ACCT closure (table 1 for breakdown of these episodes). Survival curves stratified by selected risk factors are shown in online supplemental eFigure 2.

### Risk prediction model development

A Cox proportional hazard model was fitted as described in the Statistical analysis section (see online supplemental appendix). Potential risk factors were all variables listed in table 1, with the first five variables shown prespecified for inclusion and remaining variables assessed using backwards selection. In total, 25 potential risk factors were examined across domains of criminal history, suicidality/ACCT-related, and health. Two proposed risk factors (number of case reviews, number of transfers in current prison term) could not be used as suitable data were unavailable. As the proportion of missing data among variables retained in the final model was very low (n=17, 2%), imputation was not used for the final model fitting and the complete-case dataset (n=737) was used.

The final model had nine factors. Of the risk factors that were not prespecified, four were retained in the final model: first time in custody, cutting as the method of index self-harm, previous self-harm recorded at reception screening, and length of ACCT in days. Adjusted HRs are shown in table 2. Male sex, older age and first time in custody were inversely associated with repeat suicidality during follow-up, while the presence of all other risk factors was associated with increased risk of repeat ACCT. Cutting as the method of index self-harm was partially confounded with reason for ACCT opening as it can only occur if self-harm was the reason for ACCT opening, explaining the apparent reduction in the direct effect of the latter. Longer ACCTs, once closed, were associated with higher risks of repeat suicidality.

**Table 2:** Risk factors for repeat suicidality in people in prison from multivariable survival analysis model

Risk factor	Coefficient	SE	Adjusted hazard ratio (95% CI)
Male sex	−0.4693	0.2109	0.63 (0.41, 0.95)
Age (per 5 years)	−0.1224	0.0412	0.88 (0.82, 0.96)
**Reason for ACCT opening**			
Low mood	–	–	Ref.
Threat of self-harm	0.2410	0.2360	1.27 (0.80, 2.02)
Self-harm	0.1847	0.2636	1.20 (0.72, 2.02)
Suicidal thoughts (at ACCT opening)	0.3053	0.1746	1.36 (0.96, 1.91)
Previous ACCT within 6 months before index ACCT	0.3050	0.1649	1.36 (0.98, 1.87)
First time in custody	−0.4183	0.1654	0.66 (0.48, 0.91)
Self-harm method: cutting	0.5555	0.2405	1.74 (1.09, 2.79)
Previous self-harm recorded at reception screening	0.3730	0.1745	1.45 (1.03, 2.04)
Length of ACCT			
0–1 days	–	–	Ref.
2–9 days	0.6674	0.2607	1.95 (1.17, 3.25)
10+ days	0.8666	0.2383	2.38 (1.49, 3.79)

ACCTAssessment, Care in Custody and Teamwork

### Risk prediction model performance

**Figure 1:**
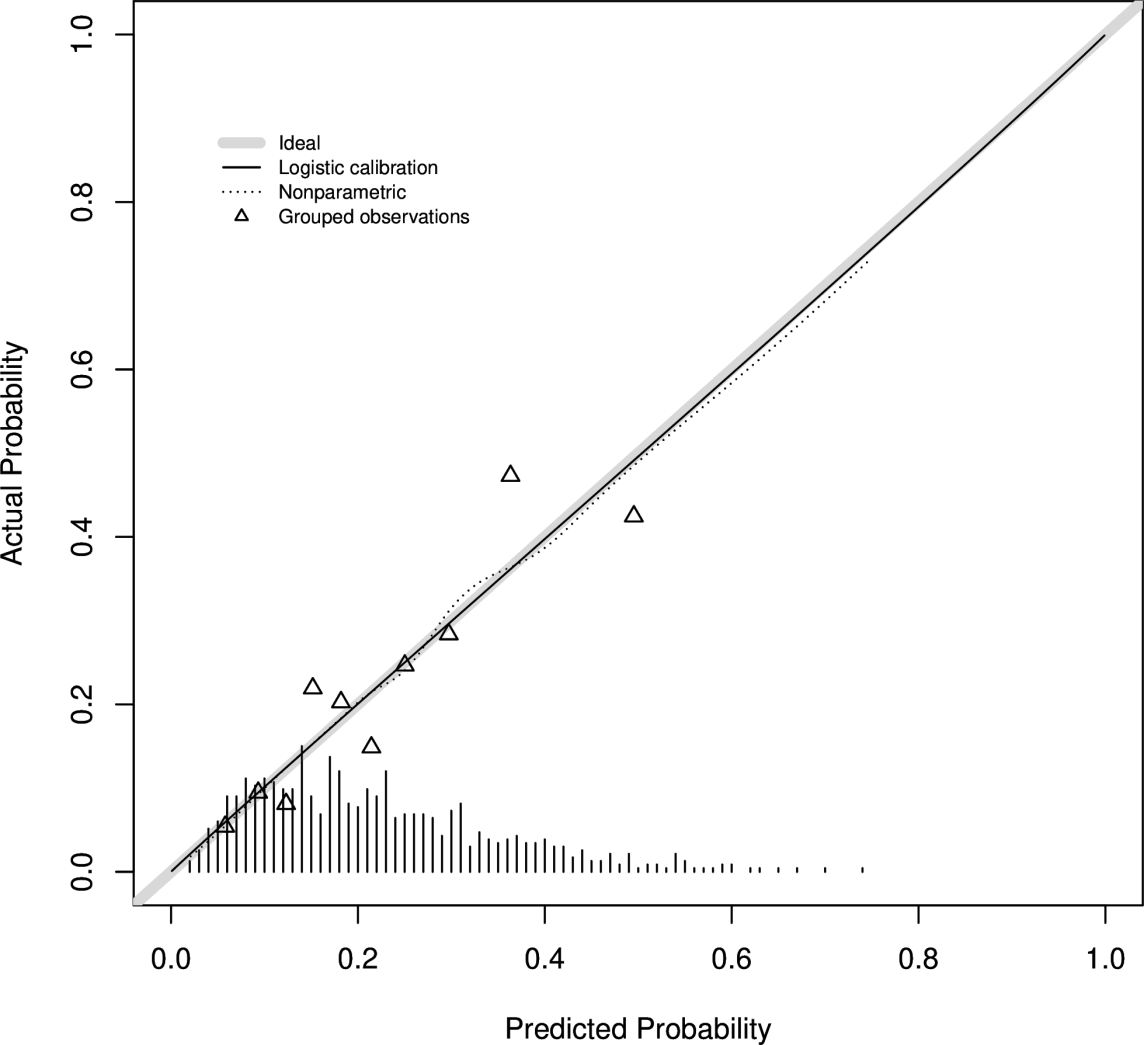
Calibration plot for development cohort for the RAPSS risk model for repeat suicidality. Calibration-in-the-large=1.00 (95% CI 0.86 to 1.17). RAPSS, Risk Assessment for people in Prison at risk of Self-harm and Suicide.

The predicted probability of repeat suicidality within 90 days of index ACCT closure was calculated for each individual in the development dataset and compared between participants who did and did not experience a repeat ACCT. Although probabilities tended to be higher in those with a repeat ACCT, there was considerable overlap in the distribution of probabilities. The apparent Harrell’s c-statistic (for time-to-event data) was 0.68 (95% CI 0.64 to 0.72), and the estimate was 0.66 after adjustment for optimism using bootstrapping. There were only minor differences between prisons in this measure (data not shown). These results are further illustrated in the receiver operating characteristic (ROC) curve for development cohort (online supplemental eFigure 3). The predictive model was associated with good calibration in a plot (figure 1).

Additionally, classification measures (sensitivity, specificity, positive predictive value and negative predictive value) were calculated for a variety of thresholds (ie, considering a predicted probability if it exceeds a given value). As no threshold was prespecified, table 3 shows performance at a range of thresholds (positivity thresholds).

**Table 3:** Model performance estimates for the RAPSS risk model for repeat suicidality across a range of thresholds

Threshold	Sensitivity	Specificity	PPV	NPV
≥ 0.15	0.87 (0.80, 0.91)	0.41 (0.37, 0.46)	0.30 (0.26, 0.34)	0.92 (0.87, 0.95)
≥ 0.2	0.71 (0.63, 0.78)	0.57 (0.53, 0.61)	0.32 (0.27, 0.37)	0.87 (0.83, 0.90)
≥ 0.25	0.58 (0.50, 0.66)	0.72 (0.68, 0.75)	0.37 (0.31, 0.43)	0.86 (0.82, 0.89)
≥ 0.3	0.45 (0.37, 0.53)	0.81 (0.77, 0.84)	0.40 (0.33, 0.48)	0.84 (0.80, 0.87)
≥ 0.35	0.34 (0.27, 0.42)	0.89 (0.86, 0.91)	0.46 (0.37, 0.56)	0.82 (0.79, 0.85)

Note: For comparison, observed prevalence of repeat suicidality across whole cohort was 0.22.

NPVnegative predictive valuePPVpositive predictive valueRAPSSRisk Assessment for people in Prison at risk of Self-harm and Suicide

### Validation study

We recruited 475 people in prison for validation. As healthcare data were unavailable for 85 people who had been transferred or released before data access was granted, the final cohort consisted of 390 people from 13 prisons (10 male and 3 female prisons). ACCTs were opened between October 2021 and March 2023, with the majority dating from mid-2022 onwards. Of the 390 people in prison, 86 (22%) had the outcome of repeat suicidality within 90 days of the index ACCT, similar to the development sample. A further 32 people in prison were subsequently excluded from the validation analysis because of missing data (see online supplemental eFigure 4 for flowchart of recruitment).

Most risk factors were distributed similarly as in the development sample (online supplemental eTable 2). Of the variables included, two variables—suicidal thoughts and first time in custody—had weaker associations with the outcome in the validation sample than in development (online supplemental eTable 3).

#### Risk model performance in external validation

The predicted probability of repeat suicidality was calculated for each individual in external validation and compared between participants who did and did not experience a repeat ACCT within 90 days. The overall c-index was 0.66 (0.60, 0.72). Classification metrics at different thresholds are presented in online supplemental eTable 3 with ROC plot shown in online supplemental eFigure 5. Using the same range of positivity thresholds as in development (online supplemental eTable 2), similar levels of predictive performance were found. Most prisons had too few outcome events to allow a comparison of performance measures between prisons.

Calibration metrics were good. Estimated calibration-in-the-large (defined as the observed number of outcome events, divided by the total expected number of outcome events based on predictions from the model) was 1.09 (95% CI 0.88 to 1.35), based on 84.8 predicted events and 78 observed events (among those for whom a prediction could be calculated, that is, after excluding those with missing predictors). The calibration plot shows good validation (online supplemental eFigure 6). There was some evidence of overprediction among individuals assessed to be at the highest levels of risk, but this was relevant to very few individuals.

## Conclusion

In this study of repeat suicidality in people in prison, we developed and validated a risk model in 1144 individuals in prisons across England. The final model, which was based on routinely collected information, included nine predictors, and its performance was good when combining discrimination, calibration and classification measures. To our knowledge, this is the largest study of repeat suicidality in people in prison internationally and also the largest to develop a risk assessment model for suicidal outcomes in prison. The information on risk factors provided some unexpected findings, which underscores the value of the methodological approach used. We have translated the final model into a simple scalable risk tool (called Risk Assessment for people in Prison at risk of Self-harm and Suicide, RAPSS).

Interpretation of RAPSS model performance requires taking into account the full range of metrics. Overall discrimination (with a c-index of 0.68 in development and 0.66 in validation) is moderate, rather than high. However, this should not be interpreted in isolation from the decision being made, alternatives and other performance measures. Notably, when combined with calibration and classification, overall performance was good. Furthermore, a key issue is the comparison. Current practice is an unstructured review of needs by a prison officer 7 days after the risk management plan (ACCT) is closed. There is no evidence if this post-ACCT needs assessment is accurate, is biased against certain subgroups or presentations, improves outcomes, or leads to identified psychosocial and healthcare needs being addressed. A review of unstructured risk assessment by clinicians for self-harm in clinical settings found poor sensitivity (at 0.31), worse than sensitivities at all thresholds (from 15% to 35%) tested for the current model.^17^ Sensitivity is an important classification measure, although influenced by threshold choice, as minimising false negatives (ie, higher sensitivities) will be more important than false positives (ie, higher specificities). Performance of non-clinician staff is likely to be worse still, which needs to be considered when interpreting these performance metrics. One other study has investigated another approach, which is using symptom checklist scores (including CORE-10, PHQ-9, BSL-23 and PriSnQUest psychiatric screen) to predict repeat self-harm at 6 months. Discrimination was poor and AUCs (area under the curve values) ranged from 0.50 (no better than chance) to 0.56.^8^

In addition to a new risk model, this study provides evidence on risk factors for repeat suicidality. Previous work has focused on any self-harm, which is typically the first self-harm episode after arrival in prison,^5^ and used case-control designs. The predictive performance of tools at this reception stage has been poor to moderate^18 19^ but may be used to screen out higher risk individuals. The current study found that female sex, younger age, previous suicidality, and cutting as a method of index self-harm were all strong risk factors for repeat suicidality. Being in custody for the first time was associated with a lower risk, which has not been reported previously as a risk factor for any or repeat self-harm in prison.^3 5^ One explanation for this unexpected finding is that people in prison for the first time may not use ACCTs for instrumental reasons, for example, changing cells, moving to a single cell or accessing certain privileges (eg, vapes). These privileges can be part of a broader therapeutic programme for someone who is on an ACCT to address unmet needs. Another risk factor for repeat suicidality reported in the current study was a previous ACCT in the prior 6 months. As some of these previous ACCTs were initiated by self-harm episodes, this is consistent with the previous suicidal behaviours being a strong risk factor. Finally, the study found that cutting was the most common method of self-harm, underscoring the role of limiting access to means as part of any suicide prevention strategy.

A further finding is that the risk factors for repeat suicidality in the current investigation were not as strong as those reported in other work for any self-harm in prison. This is particularly the case for clinical factors, such as psychiatric diagnosis, whereas in the current study, this was not found as a risk factor for repeat suicidality, either as a clinician-recorded diagnosis or using psychotropic medication as a proxy. In addition, there were many candidate predictors that were not significantly associated with repeat suicidality in the current multivariable model, including criminal history variables, ethnicity and marital status. In contrast, for any self-harm in prison, these factors have previously been shown to be important risk factors.^5^ Overall, this suggests that risk factors for repeat suicidality are distinct in two ways—first, they are mostly restricted to the current episode and past suicidality, and, second, they have smaller effect sizes than the risk factors for first self-harm in the prison setting. One explanation for this is that repeat self-harm is influenced by short-term triggers and secondary gains in custody, rather than mental health factors that are strongly associated with first self-harm episodes in prison.

The final set of nine variables in the RAPSS model are not modifiable and comprise of sociodemographic factors, information about the index ACCT (including self-harm method, reason for opening), whether there has been a previous suicide risk management (ACCT) plan, and one item based on healthcare screening on arrival to prison. We tested a range of modifiable factors, including diagnosis, abnormal liver function on blood testing (as a marker of severe alcohol misuse or injection drug use), engaging with healthcare services, and medication prescription (as a marker for underlying morbidities), but none remained independently associated with the outcome when combined in a statistical model. The lack of modifiable factors may reflect their lack of incremental value for repeat suicidality, whereas they have been found to predict incident self-harm. Furthermore, modifiability may exist at the symptom level. However, requiring new clinical information such as symptom scores will require additional resources (eg, extra interviewing by clinically trained staff) and not be scalable for most prison services. Using electronic health records to determine history of self-harm can be improved by AI models,^20^ although implementing models using machine learning approaches will not be currently feasible. One limitation of the validation is the number of outcome events was 86, whereas more than 100 is recommended—further validations should test the model in new settings (with recalibration if base rates are different). Another consideration is the model’s generalisability in light of heterogeneity of self-harm prevalence,^3^ and local validations could be considered part of an implementation process. Feasibility, operationalising probability scores and collaborative risk assessment are research priorities. We have translated the model into an online risk calculator (RAPSS; https://oxrisk.com/rapsstrial/) for research and piloting. Probability scores may lead to thresholds being applied in practice. However, these risk scores allow for more precision than a binary categorisation, different cut-offs to be applied based on the clinical setting and prison context, and more professional discretion for people whose assessed risk is close to either side of a cut-off. Whether this model addresses biases in decision-making or improves outcomes was outside the scope of this study, and can be considered in future work.

One implication of the RAPSS risk model is that it can lead to interventions that bridge the suicide risk management ACCT process to the normal prison regime. Thus, the model has a therapeutic goal if linked to preventive measures. This is a clear advance from current practice in prisons where there is no structured approach to linking the end of an ACCT to the normal regime, apart from, for example, reopening a risk management plan. This would move the current risk model (and associated RAPSS tool) away from being solely focused on prediction towards a more therapeutic role. Furthermore, the tool can underscore safety planning at the end of an ACCT because everyone has a risk score using the model—a further therapeutic aspect. Finally, the RAPSS model can potentially highlight those people in prison that need additional risk management, for example, with a review of their psychosocial needs with their designated prison officer or a routine review after a set period (eg, 1 week or month). In contrast, current approaches are not based on empirically derived models; rather they are based on subjective views of a prison officer, who may or may not involve a multidisciplinary team to further consider psychosocial needs and how they can be addressed.

In England recently, clinical guidance from NICE has recommended that risk assessment tools for suicide and self-harm should not be used.^21^ However, this guidance is based on studies conducted in community settings with older and mostly unvalidated tools, and only examined tools that act as classifiers (rather than providing probability estimates).^22^ In addition, the wider international picture needs to be considered. The US National Strategy for Suicide Prevention recommends use of suicide prediction tools, and the European Psychiatric Association has endorsed tools as adjuncts to individual clinical assessment.^23 24^ The latter is consistent with research in severe mental illness that has recommended tools augment clinical decision-making.^25^ A recent risk asssessment model (OxSET), published since NICE guidance, aslo providing probability scores, addresses concerns about accuracy (with good calibration and discrimination in external validations), prevention paradox, and allows for evidence-based and transparent decisions about resource allocation after self-harm presentations to healthcare.^26^

A key challenge for prisons to address high rates of self-harm and suicide has been to provide a model of care that addresses needs at all stages during an individual’s time in prison. This involves screening for psychiatric conditions and suicide risk on arrival,^18^ and risk management processes when someone has self-harmed or at elevated risk. The RAPSS model fills a gap in wrap-around care for suicide prevention by providing a structured approach to bridging the end of a risk management plan with return to the normal prison regime.

## supplementary material

10.1136/bmjment-2024-301280 online supplemental file 1

10.1136/bmjment-2024-301280 online supplemental file 2

## Data Availability

Data may be obtained from a third party and are not publicly available.
