# Supplementary material for: Bridging assessment and treatment for repeat suicidality in prisons: development and validation of a risk model
Source: BMJ Ment Health. 2025 Feb 27;28(1):e301280. doi: 10.1136/bmjment-2024-301280 (PMC11873324; doi:10.1136/bmjment-2024-301280)
Supplement: online supplemental file 1 [file bmjment-28-1-s001.pdf]

Appendix

	Page
eFigure 1: Flowchart for prison participation for development study	2
Prediction model formula	3
eFigure 2: Survival (Kaplan-Meier) plot of time to first repeat ACCT after index ACCT closure, by selected risk factors	4
Efigure 3: ROC plot for development cohort for the RAPSS prediction model for repeat suicidality	5
eFigure 4: Flowchart for prison participation for external validation study	6
eTable 1 2x2 table of reasons for ACCT opening by length of ACCT	
eTable 2 – prisoner characteristics of external validation sample	8-9
eTable 3: Model performance estimates for a range of positivity thresholds in the predicted probability, external validation sample	10
eFigure 5: ROC plot, for risk of repeat suicidality in the external validation cohort	11
eFigure 6: Calibration plot for external validation cohort of the RAPPS prediction model for repeat suicidality.	12
Details of candidate predictor selection	13

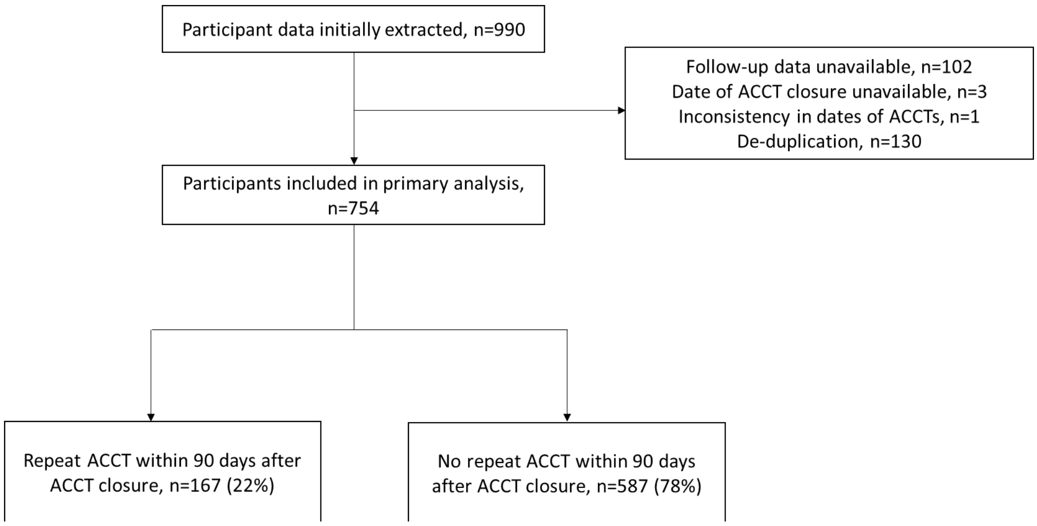

eFigure 1: Flowchart of participants in the development sample

Prediction model formula

The probability of repeat ACCT within 90 days of index ACCT closure is calculated as:

$$1 - 0.7751^{\exp(L)}$$

where

L = – 0.4693 * (Male sex)
– 0.024480 * (Age in years)
+ 0.2410 * (Reason for ACCT opening : Threat of self-harm)
+ 0.1847 * (Reason for ACCT opening : Self-harm)
+ 0.3053 * (Suicidal thoughts at ACCT opening)
+ 0.3050 * (Previous ACCT within 6 months before index ACCT)
– 0.4183 * (First time in custody)
+ 0.5555 * (Self-harm method : Cutting)
+ 0.3730 * (Previous self-harm recorded at reception screening)
+ 0.6674 * (Length of ACCT : 2-9 days)
+ 0.8666 * (Length of ACCT : 10+ days)

All variables in the above equation are coded as 0 (risk factor absent) or 1 (risk factor present), with the exception of age in years.

The conditional probability of repeat ACCT within 90 days of index ACCT closure, given no repeat ACCT has occurred within the first d days after index ACCT closure, is

$$1 - (0.7751/X_d)^{\exp(L)}$$

where the values of X_d for selected d are shown below.

d (days)	0	10	20	30	45	60
X_d	1	0.9480	0.9130	0.8793	0.8482	0.8255

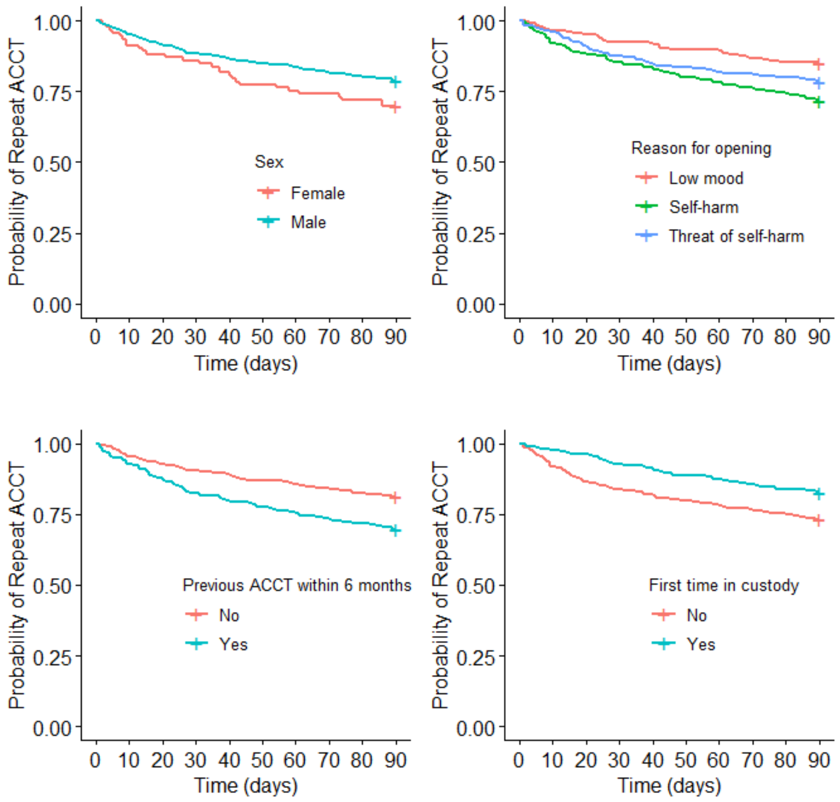

eFigure 2: Survival (Kaplan-Meier) plot of time to first repeat ACCT after index ACCT closure, by selected risk factors.

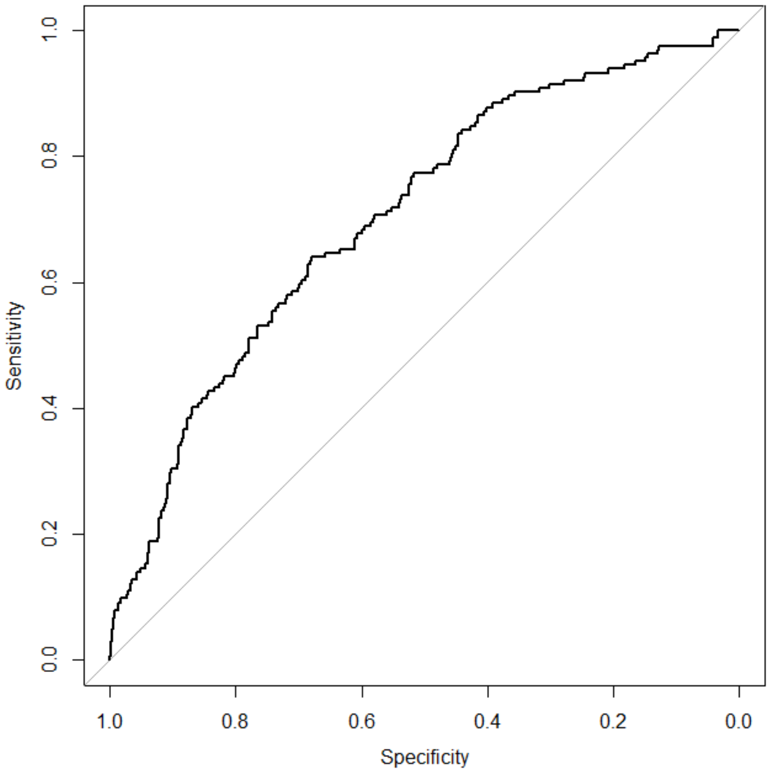

c-index 0.68 (0.64-0.72)

eFigure 3: ROC plot for development cohort for the RAPSS prediction model for repeat suicidality

	Length of ACCT		
	**0-1 days**	**2-9 days**	**10+ days**
**Low mood**	58	52	101
**Threat of self-harm**	64	70	105
**Self-harm**	67	90	147

eTable 1 – Length of an ACCT according to reasons for opening the ACCT

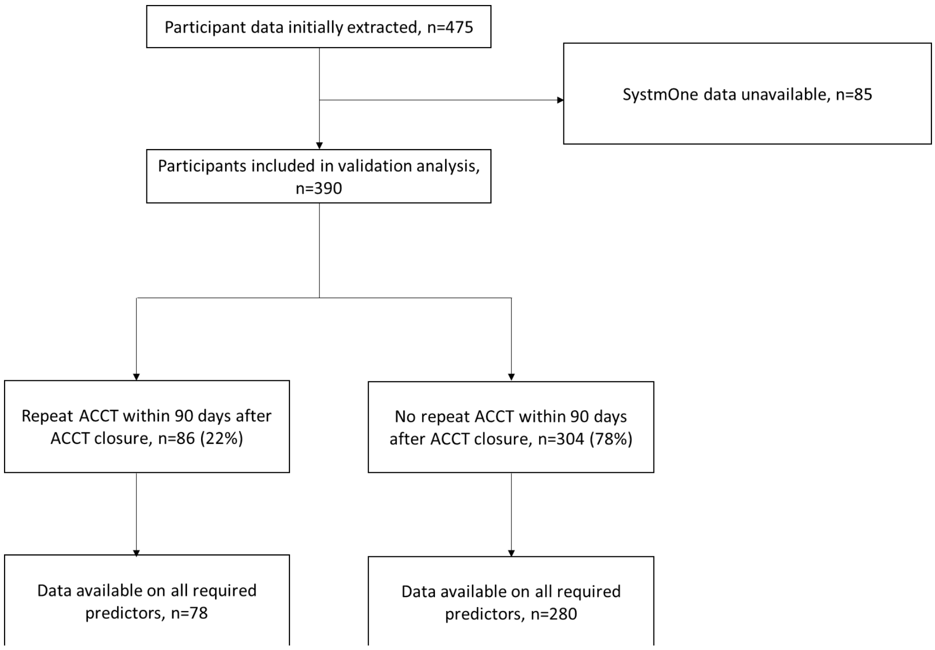

eFigure 4: Flowchart for prison participation for external validation study

Note: This sampling strategy was preferred to completely random split sampling at the individual level for development and validation, which overestimates model performance, partly as risk factor distribution and outcome prevalence will inevitably be very similar and model development will be in a smaller dataset, increasing overfitting risk.

eTable 2 – prisoner characteristics of external validation sample

Risk factor	Total (n=390)	No follow-up ACCT within 90 days (n=304)	Repeat ACCT within 90 days (n=86
**Male sex**	**305 (78%)**	**249 (82%)**	**56 (65%)**
**Age (years)**	**33 (27, 40)**	**34 (27, 42)**	**32 (28, 38)**
**Reason for ACCT opening**<br>**Low mood**<br>**Threat of self-harm**<br>**Self-harm**	 **82 (21%)**<br>**144 (37%)**<br>**162 (42%)**	 **67 (22%)**<br>**118 (39%)**<br>**117 (39%)**	 **15 (17%)**<br>**26 (30%)**<br>**45 (52%)**
**Suicidal thoughts (at ACCT opening)**	**106 (29%)**	**82 (29%)**	**24 (31%)**
**Previous ACCT within 6 months before index ACCT**	**105 (27%)**	**64 (21%)**	**41 (48%)**
Ethnicity: non-white	93 (25%)	79 (27%)	14 (16%)
Marital status: currently single	316 (89%)	249 (89%)	67 (92%)
Violent index offence	266 (69%)	210 (70%)	56 (65%)
**First time in custody**	**208 (54%)**	**162 (54%)**	**46 (53%)**
Sentence type<br>Life<br>IPP / Other†	 33 (8%)<br>94 (24%)	 29 (10%)<br>71 (23%)	 4 (5%)<br>23 (27%)
Time between ACCT closure and expected release (years)	0.8 (0, 1.7)	0.8 (0, 1.7)	1.1 (0, 1.8)
Drug screen	139 (36%)	108 (36%)	31 (36%)
Time between reception into custody and ACCT opening (days)	299 (29, 1803)	356 (30, 1959)	258 (19, 1579)
Self-harm method*<br>**Cutting**<br>Strangulation<br>Overdose<br>Other method	 **96 (25%)**<br>26 (7%)<br>20 (5%)<br>13 (3%)	 **66 (22%)**<br>20 (7%)<br>14 (5%)<br>12 (4%)	 **30 (35%)**<br>6 (7%)<br>6 (7%)<br>1 (1%)
CAREMAP completed	249 (72%)	198 (73%)	51 (68%)
Physical health / GP referral	70 (18%)	54 (18%)	16 (19%)
Mental health referral	210 (54%)	159 (52%)	51 (59%)
IDTS/DARS referral	49 (13%)	35 (12%)	14 (16%)
Raised/High risk at first case review	71 (18%)	52 (17%)	19 (22%)
Friend/family support	287 (81%)	221 (80%)	66 (86%)
Previous diagnoses‡<br>Chronic Physical Condition	 51 (13%)	 41 (13%)	 10 (12%)

Learning Disability /	46 (12%)	31 (10%)	15 (17%)
Neurodevelopmental			
Disorder	29 (7%)	22 (7%)	7 (8%)
Mental Illness	20 (5%)	14 (5%)	6 (7%)
Substance Misuse			
Current medication			
Antidepressants	188 (48%)	148 (49%)	40 (47%)
ADHD medication	10 (3%)	7 (2%)	(3%)
Antipsychotics	66 (17%)	44 (14%)	22 (26%)
Mood stabilisers	15 (4%)	10 (3%)	5 (6%)
Opioids	48 (12%)	39 (13%)	9 (10%)
Sleepers	(0%)	0 (0%)	(1%)
Pain relief	47 (12%)	35 (12%)	12 (14%)
Previous psychotropic medication (6 months before ACCT)	127 (33%)	92 (30%)	35 (41%)
Abnormal liver function enzymes	81 (21%)	62 (20%)	19 (22%)
Engaging with primary care	346 (89%)	271 (89%)	75 (87%)
Engaging with mental health care	303 (78%)	236 (78%)	67 (78%)
**Previous self-harm recorded at reception screening**	**247 (63%)**	**178 (59%)**	**69 (80%)**
In community	82 (21%)	63 (21%)	19 (22%)
In custody	46 (12%)	34 (11%)	12 (14%)
In community and in custody	118 (30%)	80 (26%)	38 (44%)
**Length of ACCT**			
**0-1 days**	**70 (18%)**	**61 (20%)**	**9 (13%)**
**2-9 days**	**115 (29%)**	**91 (30%)**	**24 (28%)**
**10+ days**	**205 (53%)**	**152 (50%)**	**53 (62%)**

Table shows n (%) or median (inter-quartile range). Percentages calculated among individuals with available data.

Variables in **bold face** were used in the risk prediction model.
* Among those with self-harm as the reason for ACCT opening. Individuals may have more than one reason for opening recorded. † Includes Imprisonment for Public Protection (IPP) and other non-life sentences for which no release date is specified. ‡ Chronic Physical Condition and Learning Disability / Neurodevelopmental Disorder: lifetime diagnosis. Mental Illness and Substance Misuse: diagnosis within 12 months prior to ACCT closure.
Variables with missing data: Reason for ACCT opening: 2, Suicidal thoughts: 27, Ethnicity: 12, Marital status: 36, Index offence: 5, First time in custody: 4, Sentence type: 2, Time between ACCT closure and expected release: 4, Time between reception into custody and ACCT opening: 26, CAREMAP completed: 42, Physical health/GP referral: 2, Mental health referral: 1, IDTS/DARS referral: 1, Risk at first case review: 2, Friend/family support: 36, Previous psychotropic medication: 2, Abnormal liver function enzymes: 1, Previous self-harm location: 1.

The validation contained a slightly lower proportion of males due to sampling more female prisons. A lower proportion of participants reported suicidal thoughts at ACCT opening, average time to expected release was shorter, and fewer were classified as diagnosed with a physical health condition.

Threshold	Sensitivity	Specificity	PPV	NPV
**≥ 0.15**	0.87 (0.84, 0.94)	0.33 (0.27, 0.39)	0.27 (0.21, 0.32)	0.90 (0.83, 0.95)
**≥ 0.2**	0.73 (0.62, 0.82)	0.51 (0.45, 0.57)	0.30 (0.23, 0.37)	0.87 (0.81, 0.92)
**≥ 0.25**	0.59 (0.47, 0.70)	0.69 (0.63, 0.74)	0.35 (0.27, 0.43)	0.86 (0.81, 0.90)
**≥ 0.3**	0.46 (0.35, 0.58)	0.78 (0.73, 0.83)	0.37 (0.27, 0.47)	0.84 (0.79, 0.88)
**≥ 0.35**	0.32 (0.22, 0.44)	0.86 (0.82, 0.90)	0.40 (0.28, 0.53)	0.82 (0.77, 0.86)

eTable 3: Model performance estimates for a range of positivity thresholds in the predicted probability, external validation sample

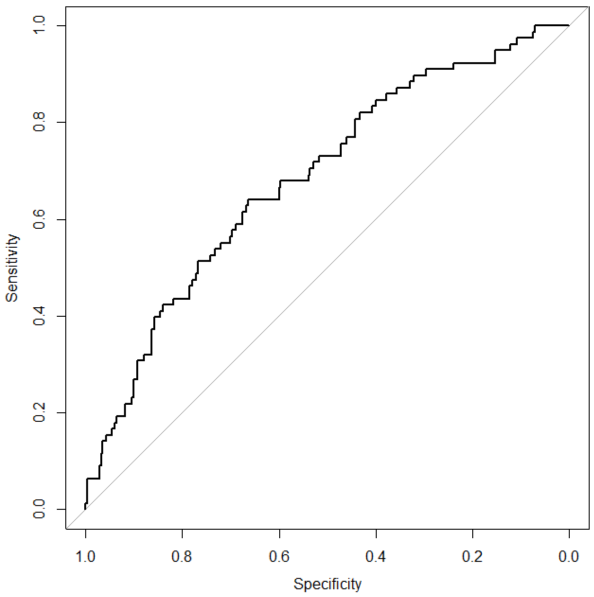

c-index 0.66 (0.60-0.72)

eFigure 5: ROC plot, for risk of repeat suicidality in the external validation cohort

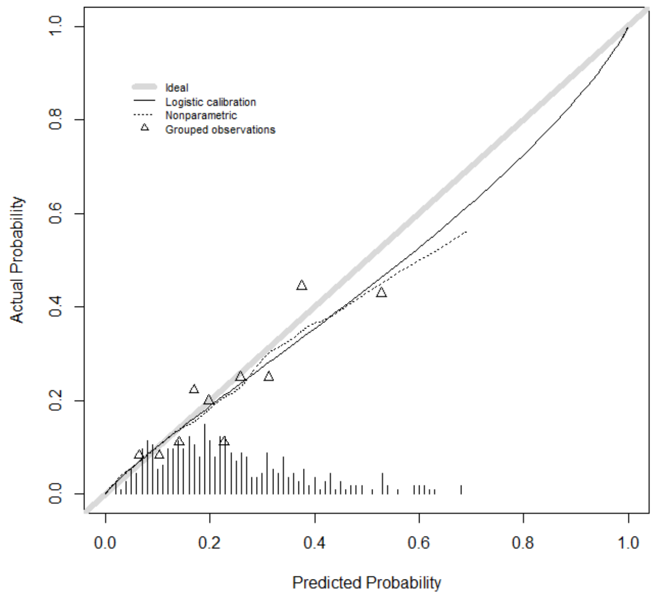

eFigure 6: Calibration plot for external validation cohort of the RAPPS prediction model for repeat suicidality.

Candidate predictor selection

One group contained candidate factors to be retained in the model because of a clear association with repeat self-harm, which was based on reviewing the literature. The other group contained the remaining factors, selected using backward stepwise selection (5% significance level), where the evidence from previous studies was less certain or inconsistent. All factors were available at the index assessment. We planned to investigate the effect of time-varying risk factors, but were unable to do this because most risk factors were only available at one time point (the index ACCT) and many were fixed (static) risk factors.

Data were collected at each prison site by a designated research assistant who collected from the paper copy of the ACCT documentation and the prison records system (C-NOMIS). Remote data collection for SystmOne information was collected by remote desktop access to each of the sites with a team of researchers who were responsible for matching the prison number of the person in custody with the NHS identification number.
