# Supplementary material for: Bridging assessment and treatment for repeat suicidality in prisons: development and validation of a risk model
Source: BMJ Ment Health. 2025 Feb 27;28(1):e301280. doi: 10.1136/bmjment-2024-301280 (PMC11873324; doi:10.1136/bmjment-2024-301280)
Supplement: online supplemental file 2 [file bmjment-28-1-s002.pdf]

Development and validation of a risk assessment tool for self-harm in prisoners (RAPSS) – Statistical Analysis Plan

Version 1.2

Date: 4/7/2023

Version History

Version:	Version Date:	Changes:
0.1	23/7/22	Original version
0.2	13/11/22	Update variable list
0.3	21/11/22	Update background & variable list
0.4	13/12/22	Update variable list. Add provisional priority grouping of risk factors. Update statistical analysis text.
0.5	15/1/23	Update variable list, clarify outcome and index ACCT definitions.
0.6	30/1/23	Add one variable to variable list
0.7	22/5/23	Clarification of diagnosis risk factors and outcome definitions
1.0	19/6/23	Set as v1.0
1.1	28/6/23	Amendment to timepoint for definition of diagnosis risk factor
1.2	4/7/23	Removal of self-harm during ACCT risk factor as a result of amendment of definition; clarify length of ACCT variable

Study Summary

Design: Retrospective multi-centre cohort study.

Participants: Male and female prisoners, aged 18+, in selected category A-C prisons in England who have been managed by the Assessment, Care in Custody and Teamwork (ACCT) process in response to concerns of being at risk for self-harm or suicide.

Data sources: Three linked data sources: ACCT records, SystmOne medical records and Nomis (a central prison database).

Study period: Included participants had ACCTs closed prior to June 2022, with a minimum of three months of follow-up per participant.

Risk factors: A range of variables including demographics characteristics, variables indicative of mental health at opening and closure of the ACCT, history of self-harm, variables relating to history of and reasons for incarceration, variables indicative of health status and medication use.

Primary outcome: ACCT reopening within 3 months following closure of the ACCT. The target sample size was 750, anticipating that a 20% event rate would yield 150 outcome events within 3 months.

Outputs: The main objective is to produce models that estimate outcome probabilities within 3 months, with appropriate measures of predictive accuracy.

These models will be used to obtain scoring systems for the calculation of risk to be used prospectively at the time of ACCT closure.

1. Study background

Rates of self-harm in prisoners are high in absolute and relative terms. In 2021, there were 54,027 reported incidents of self-harm in 11,292 prisoners, a rate of 688 per 1,000 prisoners. This has increased from the period of 2011-2017, where the annual rate was below 500 per 1,000 prisoners. Over the same period, there were more than 2,400 hospital attendances as a result of self-harm incidents, a rate substantially higher than in community-based persons. Self-harm is the strongest risk factor for suicide in custody and on release, which are both elevated compared to population controls.

The ACCT process (Assessment, Care in Custody and Teamwork) was introduced to improve risk management process (Prison Service Instruction 64/2011). Previous NIHR-funded research showed that 28% of prisoners self-harmed in the 6 months following closure of the ACCT document (Horton, 2014; Horton 2018). This research concluded that a particular evidence gap was the lack of a structured approach for the assessment of risk of recurrent self-harm on ACCT closure, and that existing tools and models were not adequate. Their research also highlighted the potential importance of novel modifiable risk factors, that if studied in larger samples, could inform identification of needs, assessment and risk management.

A particular problem identified was the lack of a structured approach for the assessment of risk of recurrent self-harm on ACCT closure. This research is necessary to improve the process by which risk can be assessed and stratified after an episode of self-harm. Current clinical practice in England and Wales, and also internationally, lacks reliable and valid structured approaches to stratify risk of recurrent self-harm, link higher risk prisoners to appropriate care pathways, and more efficiently allocate limited resources.

This Statistical Analysis Plan relates to a retrospective cohort study for the development of a risk assessment tool for self-harm within three months in prisoners at the point of ACCT closure. The wider project contains further substudies: a qualitative investigation into acceptability of the risk prediction tool to relevant stakeholders, and a prospective validation study of the performance of the risk prediction tool in an external validation sample.

2. Statistical Analysis

Available variables, and the data sources used to collect them, are listed in Appendix 1.

The main statistical analysis method used will be Cox proportional hazards regression for survival analysis, using time to first ACCT reopening following the closure of the index ACCT as the primary outcome and a set of pre-specified candidate predictors (see separate subsection below) as covariates. In the event that the goodness-of-fit of the Cox model is inadequate, other more suitable survival analysis models, including parametric survival models, will be explored.

We will further extend the basic model to allow for possible time-varying effects of risk factors (Thomas and Reyes, 2014, Section 2.3) and/or to allow dynamic prediction using time-varying covariates (Van Houwelingen and Putter, 2012) using the 'dynpred' R package, to allow the risk to be re-estimated at the time of post-ACCT review (which occurs 7 days after closure in older ACCTs and 6 weeks after closure in newer ACCTs). This may require refitting of the model to incorporate data that is available at a second baseline (the post-ACCT review) that was unavailable at the first baseline (closure of the original ACCT).

The primary outcome is self-harm and/or substantially elevated risk or concern of future self harm that leads to a new ACCT being opened, within 3 months following ACCT closure, among prisoners who remained in custody at the time of data collection. Model fitting will be performed using follow-up data within 3 months of follow-up. Risk estimates will use the 3-month event horizon and model performance measures will be calculated in relation to this time point. Risk estimates will also be expressed conditional on the length of the post-ACCT period that has already elapsed, to allow for the likely non-linear decline in risk over time, the initial 30 days after ACCT closure being a particular high-risk period for self-harm. The distribution of risk estimates observed will guide a decision about a probability threshold indicative of high risk, although performance at a range of thresholds will be presented.

Individuals for whom no outcome event has occurred at the time of last follow-up will be considered as right-censored at that time. This includes those who have left prison, for whom the release date is recorded.

Each individual has an 'index ACCT' that defines the time point at which risk factor variables are available, and the closure of this ACCT is interpreted as time 0 for defining subsequent folow-up times. In the case that more than one ACCT from the same individual is available as an index ACCT, the most recent of these that has substantially complete risk factor data will be used in the primary analysis. A sensitivity analysis will be carried out to check that the selection of this index ACCT does not have an undue influence on results.

Covariates that have more than 30% missing data will be excluded. Missing data on covariates with at most 30% missing data will be imputed via multiple imputation (with twenty imputations) using a regression model that uses other risk factors and the outcome variables as explanatory variables (Sterne et al., 2009). Estimates of coefficients in the final prediction rule will be combined across imputations, using standard methodology (Barnard and Rubin, 1999). It is possible that the variable selection procedure would give different sets of variables in different imputations of the data-set; for this reason, the 'RR' method described by Wood et al. (2008) will be used. In this method,

summary estimates are computed by combining information across all imputed data-sets at each stage as part of a single variable selection process.

Internal validation will be assessed using bootstrapping to assess the predictive accuracy of the model (Harrell et al., 1996). Predictive accuracy will be summarised using several summary measures, including the concordance index (Harrell et al., 1982) and estimates of sensitivity and specificity corresponding to different values of a risk threshold. The proportions of predicted and observed events at different levels of predicted probability will be compared using a calibration plot.

Data will be collected from multiple prison sites (expected to be 6 different prisons in England). The main model will be fitted using data from all sites combined. Performance measures will also be presented stratified by site in order to identify whether there are any major between-site differences that may impact on performance in an external validation sample.

This analysis plan does not incorporate an external validation step as it is planned to conduct a separate external validation substudy, using data from a different selection of prisons, concurrently with the analysis in the model development stage.

Risk factors

Risk factors will be considered in two groups, of differing levels of priority. The 'List of risk factor variables' table in the Appendix specifies the group to which each variable is assigned.

Group 1 consists of variables that will be included in the statistical model regardless of statistical significance. These include demographic characteristics that it is necessary to include to ensure the model has face validity, and other risk factors suspected on the basis of previous research or subject-matter judgment to be associated with a self-harm outcome.

Group 2 consists of variables likely to show an association with outcomes but which are not required to be included to achieve face validity. The model will use a backwards stepwise selection procedure to determine whether to retain these variables in the model, with Group 1 variables always retained and Group 2 variables sequentially rejected in order of p-value until no group 2 variables remain that have p-values greater than 0.1.

This strategy of risk factor adjustment recognises that the final model must demonstrate face validity, whilst simultaneously allowing the inclusion of additional risk factors if they show an association with outcome variables. The variables are considered in two groups in this way to recognise that a parsimonious model is preferable and easier to use in practice, provided that it has acceptable predictive ability.

References

Barnard J, Rubin DB. Small-sample degrees of freedom with multiple imputation. Biometrika 1999; 86: 948-955.

Harrell FE, Califf RM, Pryor DB, Lee KL, Rosati RA. Evaluating the yield of medical tests. Journal of the American Medical Association 1982; 247: 2543-2546.

Harrell FE, Lee KL, Mark DB. Multivariable prognostic models: Issues in developing models, evaluating assumptions and adequacy, and measuring and reducing errors. Statistics in Medicine 1996; 15: 361-387.

Sterne JAC, White IR, Carlin JB, Spratt M, Royston P, Kenward MG, Wood AM, Carpenter JR. Multiple imputation for missing data in epidemiological and clinical research: potential and pitfalls. BMJ 2009; 338: 157-160.

Thomas, L. and Reyes, E.M. (2014) Tutorial: Survival Estimation for Cox Regression Models with Time-Varying Coefficients Using SAS and R. Journal of Statistical Software, 61(1).

Van Houwelingen H, Putter H. Dynamic prediction in clinical survival analysis. CRC Press: 2012.

Wood AM, White IR, Royston P. How should variable selection be performed with multiply imputed data? Statistics in Medicine 2008; 30: 3227-3246.

Appendix 1: List of variables and data sources

Sex (based on single-sex prison in which prisoner is incarcerated)

<u>ACCT records</u>
Date ACCT open & closed
Reason for ACCT
Method of self-harm
Current suicidal thoughts or intentions
Number of case reviews
Level of risk at first review
CAREMAP actions completed
Referred to GP or mental health services
Friends/family support
Date of post-closure review (ACCT v5 the typical post-closure period is 7 days, ACCT v6 is 6 weeks)
Issues resolved in post-closure

<u>Nomis</u>
Expected release date
Date of first reception for this period in custody
Drug screen
Index offence
Number of transfers in current prison term
First time in custody
Dates of previous and follow-up self-harm incidents
IDTS (Integrated Drug Treatment System) / DARS (Drug and Alcohol Rehabilitation Services) referral

<u>SystmOne</u>
Age
Ethnicity
Marital status
Current medication
Previous psychotropic medication in last 6 months
Abnormal liver function enzymes
Engaging with primary care or prison mental health services
Previous self-harm at reception screening
Previous diagnoses

Variable	Source	Group	Type	Notes
**Date variables used to define risk factors and outcomes**				
Date open & closed	ACCT	-	Date	Used to define length of ACCT (see below)
Dates of previous ACCTs	Nomis	-	Date	Used to calculate measures of history of ACCT opening before the index ACCT
Expected release date	Nomis	-	Date	Used to define time until expected release (see below)
First reception for this period in custody	Nomis	-	Date	Used to calculate time between reception in custody and ACCT opening (see below)
First reception at current prison	Nomis	-	Date	Used to calculate time between reception at current prison and ACCT opening (see below)
Follow-up ACCT reopening	Nomis	-	Date	Primary outcome is time between ACCT closure and first subsequent ACCT reopening
**Risk factor variables**				
Sex	-	1	Binary	Prisons are single-sex so prison site indicates sex of prisoner. Coded as Male or Female.
Reason for ACCT opening	ACCT	1	Categorical	Categorised as self-harm; threat of self-harm; low mood (including all other reasons for ACCT opening)
Method of self-harm	ACCT	2	Categorical	Categorised as cutting; strangulation; overdose; other method; not applicable (no self-harm)
Current suicidal thoughts or intentions	ACCT	1	Binary	At ACCT opening. Categorised as Yes or No.
Number of case reviews	ACCT	2	Discrete or categorical	
Level of risk at first case review	ACCT	2	Binary	Refers to first case review after ACCT opening. Categorised as ‘High’ or ‘Low’ (with the category ‘Raised’ used in one version of the ACCT document grouped with ‘High’).
CAREMAP actions completed	ACCT	2	Binary	Refers to first case review after ACCT opening.

				Categorised as Yes or No.
Referred to GP	ACCT	2	Binary	Categorised as Yes or No
Referred to mental health	ACCT	2	Binary	Categorised as Yes or No
Friends/family support	ACCT	2	Binary	Categorised as Yes or No
Drug screen	Nomis	2	Binary	At start of custody. Categorised as Yes (positive) or No (no test recorded).
Index offence	Nomis	2	Binary	Categorised as 'Violent' or 'Non-violent' index offence
Number of transfers in current prison term	Nomis	2	Discrete or categorical	
Time until expected release	Nomis	2	Continuous	Calculated as time between ACCT closure date and expected release date. If included in model, must be used alongside life sentence and IPP variables below.
Life sentence	Nomis	2	Binary	Categorised as Yes or No
IPP (imprisonment for public protection) sentence	Nomis	2	Binary	Also includes other non-life sentences for which an expected release date is not applicable. Categorised as Yes or No.
First time in custody	Nomis	2	Binary	Categorised as Yes or No
Time since first reception in custody	Nomis	2	Continuous or categorical	
IDTS/DARS referral	Nomis	2	Binary	Integrated Drug Treatment System or Drug and Alcohol Rehabilitation Services referral. Categorised as Yes or No.
Previous ACCT within 6 months prior to ACCT opening	Nomis	1	Binary	Categorised as Yes or No
Age	SystmOne	1	Continuous	Measured at time of ACCT closure
Ethnicity	SystmOne	2	Binary	Categorised as non-white or white
Marital status	SystmOne	2	Binary	Categorised as 'Currently single' (single / widowed / divorced / separated) or 'Not currently single'
Current medication	SystmOne	2	Categorical	Categorised by medication class (antidepressants, ADHD medication, antipsychotics, mood stabilisers, opioids, sleepers, pain relief).
Previous psychotropic medication in last 6 months	SystmOne	2	Binary	Categorised as Yes or No

Abnormal liver function enzymes	SystmOne	2	Binary	Categorised as Yes or No
Engaging with primary care	SystmOne	2	Binary	Categorised as Yes or No
Engaging with mental health services (MHIRT)	SystmOne	2	Binary	Categorised as Yes or No
Previous self-harm at reception screening	SystmOne	2	Binary	Categorised as Yes or No
Previous diagnoses:<br>• Learning disability or neurodevelopmental disorder<br>• Mental illness<br>• Substance misuse<br>• Chronic physical illness	SystmOne	2	Binary	Each categorised as Yes or No<br>Learning disability or neurodevelopmental disorder and chronic physical illness variables refer to lifetime diagnosis (any time before ACCT closure).<br>The other diagnoses refer to diagnoses made within 1 year prior to ACCT closure.<br>Substance misuse includes both alcohol and drug/other substance misuse.
Length of ACCT (days)	ACCT	2	Categorical	0-1, 2-9, 10+
**Variables available at post-ACCT review**				
Date of post-closure review	ACCT	-	Date	Date of post-closure review. For ACCT v5 the usual post-closure period is 7 days, for ACCT v6 is 6 weeks.
Issues resolved in post-closure review	ACCT	2	Binary	Refers to post-ACCT closure review. Categorised as Yes or No (with ‘Partially’ classified as No).
